# The impact of orientation filtering on face-selective neurons in monkey inferior temporal cortex

**DOI:** 10.1038/srep21189

**Published:** 2016-02-16

**Authors:** Jessica Taubert, Valerie Goffaux, Goedele Van Belle, Wim Vanduffel, Rufin Vogels

**Affiliations:** 1 Face Categorization Lab, University of Louvain, Louvain-La-Neuve 1348, Belgium; 2 Laboratorium voor Neuro- en Psychofysiologie, KU Leuven, Leuven 3000, Belgium; 3 MGH Martinos Center, Charlestown, MA 02129, USA; 4 Harvard Medical School, Boston, MA 02115, USA

## Abstract

Faces convey complex social signals to primates. These signals are tolerant of some image transformations (e.g. changes in size) but not others (e.g. picture-plane rotation). By filtering face stimuli for orientation content, studies of human behavior and brain responses have shown that face processing is tuned to selective orientation ranges. In the present study, for the first time, we recorded the responses of face-selective neurons in monkey inferior temporal (IT) cortex to intact and scrambled faces that were filtered to selectively preserve horizontal or vertical information. Guided by functional maps, we recorded neurons in the lateral middle patch (ML), the lateral anterior patch (AL), and an additional region located outside of the functionally defined face-patches (CONTROL). We found that neurons in ML preferred horizontal-passed faces over their vertical-passed counterparts. Neurons in AL, however, had a preference for vertical-passed faces, while neurons in CONTROL had no systematic preference. Importantly, orientation filtering did not modulate the firing rate of neurons to phase-scrambled face stimuli in any recording region. Together these results suggest that face-selective neurons found in the face-selective patches are differentially tuned to orientation content, with horizontal tuning in area ML and vertical tuning in area AL.

The speed and ease with which primates detect, categorize and discriminate faces belies the complexity of the computations being performed by the brain. Advanced neuroscientific techniques have recently uncovered a network of interconnected, functionally-defined, areas (or ‘patches’) in monkey inferior temporal (IT) cortex that are selectively activated by face stimuli^1^,^2^,^3^. Subsequent studies of selectivity at the single neuron level have indicated a division of labor within this system whereby the more posterior patches are potentially involved in earlier stages of face perception than the more anterior patches that are thought to be more likely recruited for subordinate discrimination tasks^4^,^5^,^6^. For instance the anterior face patch known as AM has been recently implicated in the discrimination of faces belonging to different individuals (i.e. face identification^7^,^8^). Despite these advances, however, the exact nature of the visual information being processed in each of these discrete face patches remains unresolved.

Meanwhile, emerging psychophysical^9^,^10^,^11^,^12^ and electrophysiological^13^ evidence in humans has pointed to the importance of orientation content as a determinant of face processing in the human brain. For example, numerous behavioral studies have reported that information about the structure of a face, the presumed basis for face detection and identification, is carried primarily by horizontal orientation content^9^,^10^,^11^. That said, it is also possible that different orientation ranges support different aspects of face perception (for example different emotional expressions^14^). A more recent investigation of functional brain activity in humans has confirmed that at least two face-selective areas are more strongly activated by horizontal-passed faces than vertical-passed faces^15^. This sensitivity to horizontal orientation content in the face processing system was not inherited from primary visual cortex. The strong relationship between orientation content and human face processing implies that orientation filtering is a useful tool for examining functional specialization within the face processing system in rhesus monkeys.

In this paper, our first aim was to test whether face-selective neurons show the advantage for the horizontal orientation content as is observed in humans using a technique known as orientation filtering. Second, we assessed whether face-selective neurons have the same response to horizontal- and vertical-passed faces, regardless of their anatomical location within monkey IT cortex. Face-selective neurons have been reported outside the fMRI-defined network of patches in monkey IT^16^ but it is not yet known whether they serve a distinct function to neurons comprising the network or not (but see^17^,^18^,^19^). To address this question we measured the responses of neurons in three different areas of monkey IT, two face-selective patches that were localized using fMRI^18^,^20^ and an expanse of cortex in between these two patches that was not correlated with an increased hemodynamic response to faces (CONTROL). Also of interest was the comparison between the two face-selective patches, the middle-lateral face patch (ML) and the anterior-lateral face patch (AL), because they are thought to make different contributions to face perception^2^,^4^,^5^,^6^ and, thus, might differ in their preference for orientation content in faces.

In the first experiment (hereby referred to as the ‘Best Identity Experiment’) we tested each neuron with an effective face identity after image content had been restricted to horizontal or vertical information. We also investigated whether any preference for horizontal or vertical orientation information at the level of the single neuron was dependent on the face stimuli being presented upright. Picture-plane rotation (or inversion) is a manipulation known to mediate behavior towards faces^21^, as well as BOLD^22^, and single cell responses^18^,^19^,^23^ in the face-selective cortical system. Additionally, it has been recently shown that turning a face upside down has a differential impact on faces that have been filtered to preserve either horizontal or vertical information^10^,^12^,^13^,^15^. Thus, any preference for one filtering condition over another was not expected to survive picture-plane rotation. In this experiment we held stimulus position constant, relative to fixation, which means the position of the eyes would change from the upper visual field in the ‘upright’ conditions to the lower visual field in the ‘inverted’ conditions (for evidence of position tolerance for the inversion effect at the single cell level see^18^). However, such a change in visual field position could not explain differential sensitivity to orientation content in this experiment because the position of the facial features was stable across the orientation filtering conditions. We also included phase-scrambled faces to determine whether the impact of orientation filtering on the response of face-selective neurons was contingent on the presence of a face or evidence of general orientation tuning.

In the second experiment (hereby referred to as the ‘Multiple Identity Experiment’) we did not select an effective face identity for each neuron. Rather we tested every face-selective neuron with 10 different face identities in three experimental conditions: (1) full spectrum, (2) horizontal-passed, or (3) vertical-passed. In addition to ruling out stimulus-selection as a contributing factor, the second experiment was a critical extension of the first because it allowed us to measure the difference in the average firing rate between full spectrum (unfiltered) faces and their orientation-filtered counterparts.

## Results

**Figure 1:**
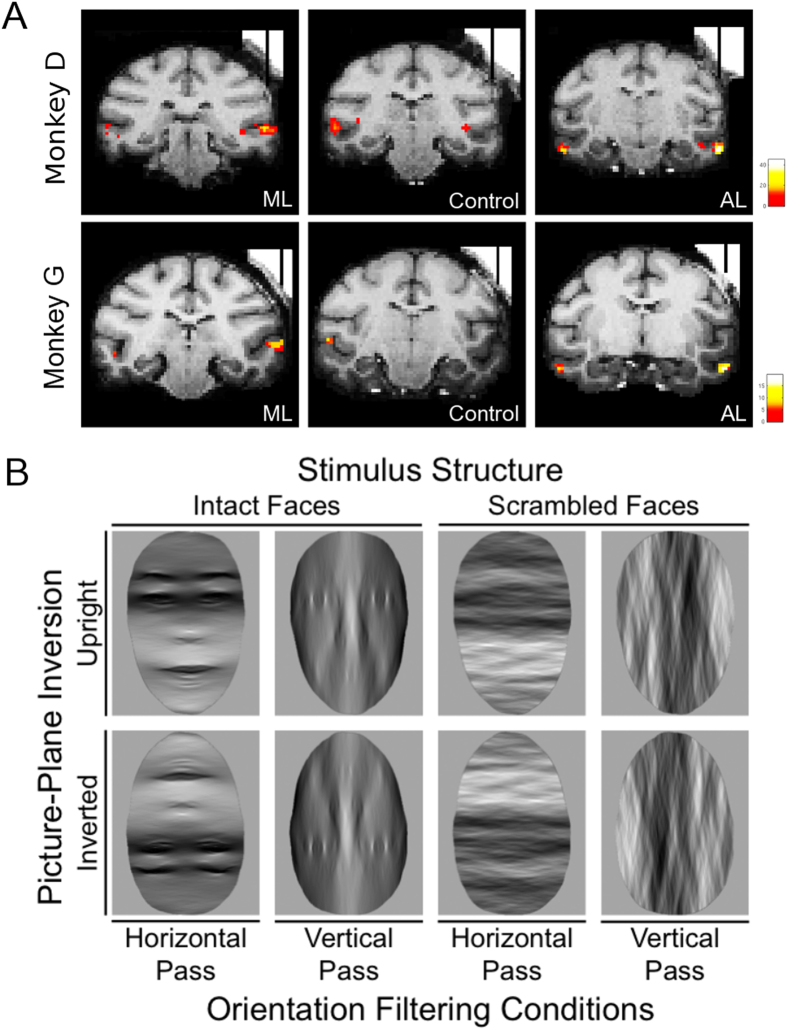
Recording locations and Experimental Stimuli. (**A**) Top row: Recording regions in Monkey D - From left to right, area ML, CONTROL and AL. Bottom row: Recording regions in Monkey G - From left to right, area ML, CONTROL and AL. In all cases, MION activation is superimposed on a high resolution anatomical scan obtained with tungsten markers positioned in the recording chamber grid. The t-maps are thresholded at p < 0.05 (Family-Wise Error), corresponding to a t > 4.9. (**B**) An illustrative example (i.e. a single identity) of the experimental conditions (Structure x Picture-Plane Rotation x Orientation Filter) used in the Best Identity Experiment.

After the fMRI localizer^18^,^19^ (see Methods) we surgically implanted a plastic recording chamber that would allow us to access ML, CONTROL and AL in the right hemisphere of two male rhesus monkeys (see Fig. 1A). To examine whether face-selective neurons responded differentially to faces that had been filtered for orientation content, we subsequently ran two experiments while recording single neuron responses in these three recording regions. During recording sessions the monkeys were awake and fixating on a central fixation point for the entire length of a trial (900 ms; see Methods). In all experiments, trials were initiated by fixation, after which there was a 300 ms fixation period before the visual stimulus was presented in the center of the screen for 300 ms. After stimulus offset, there was a further 300 ms period before the subject received a liquid reward.

All statistical analyses were performed on the net firing rate (baseline subtracted; see Methods) of visually responsive neurons unless otherwise stipulated. To ensure all neurons were ‘face-selective’, only neurons with a face-selectivity index^16^ (FSI; see Methods) greater than 0 were further analyzed^16^.

### Best Identity Experiment

In this experiment we first selected an effective face identity for each neuron. This selection procedure allowed us to include a large number of conditions in the Best Identity Experiment designed to test a neuron’s sensitivity to, both, orientation filtering and picture-plane rotation. The manipulation of the picture-plane (i.e. stimulus inversion) was included in the design because it has been shown to selectively disrupt the behavioral signature of face perception without transforming or removing any low-level image properties^24^,^25^. Moreover, recent empirical findings have indicated that turning faces upside down selectively impairs the processing of horizontal-passed faces in behavioral and neural output^9^,^10^,^11^,^12^,^13^,^14^,^15^.

The best face was presented in four intact face conditions (upright/horizontal-passed, inverted/horizontal-passed, upright/vertical-passed and inverted/vertical-passed) and four corresponding scrambled face conditions (for examples of stimuli see Fig. 1B). These eight conditions were repeated at least eight times per neuron in pseudo-random order.

Since the effect of orientation filtering may vary during the course of the response, net responses were computed for an “early time window” and a “late time window” (*early time window,* 50–200 ms relative to stimulus onset; *late time window*, 200–350 ms relative to stimulus onset). Separately for each recording region (ML, AL and CONTROL), we analyzed the data in the early time window, first, in a 2 × 2 × 2 repeated-measures ANOVA. Stimulus Structure (intact faces vs. scrambled faces), Orientation Filter (horizontal-passed vs. vertical-passed), and Picture-Plane Rotation (upright faces vs. inverted faces) were entered into these analyses as within-neuron factors. We expected larger responses in the intact face condition compared to the scrambled face condition (a main effect of Stimulus Structure), as well as a main effect of Picture-Plane Rotation (upright faces >inverted faces). The research questions regarding orientation filtering would be addressed by the main effect of Orientation Filtering, together with any interactions between Orientation Filtering and the other factors (i.e. Stimulus Structure and/or Picture-Plane Rotation). Evidence of an interaction was followed up with a series of pair-wise comparisons. The same ANOVA was performed on the responses the data in the late time window.

To examine whether a neuron’s FSI could predict its preference for horizontal-passed faces in the early or late time window, we calculated an Orientation Filtering Index (OFI) as (mean net response to upright horizontal-passed face – mean net response to upright vertical-passed face)/(|mean net response to upright horizontal-passed face |+| mean net response to upright vertical-passed face|). OFI values range between −1 and 1 and were calculated in such a way that values greater than 0 indicate a greater response to horizontal-passed faces than vertical-passed faces (and values less than 0 indicate a greater response to vertical-passed faces than horizontal-passed faces). A separate OFI was computed for each neuron’s early and late time windows.

### Area ML

**Figure 2:**
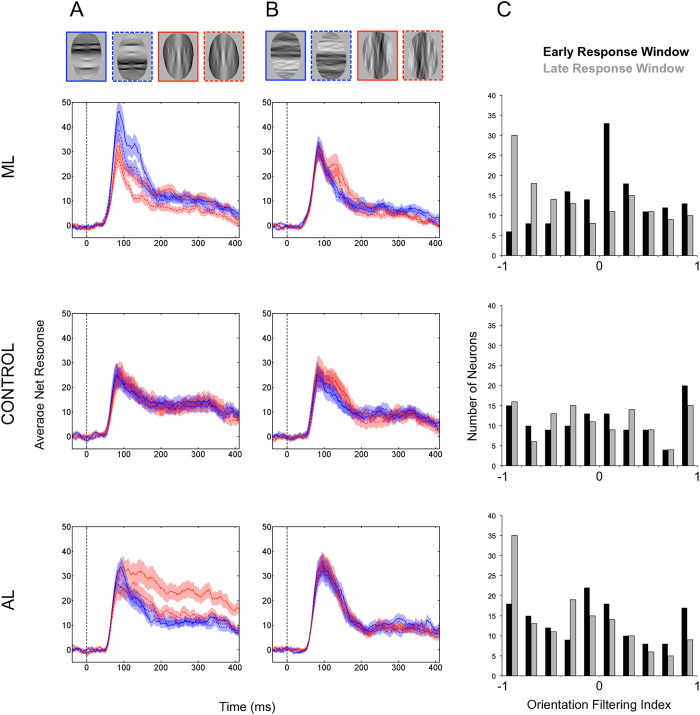
Results from the Best Identity Experiment. (**A**) Smoothed peristimulus time histograms (PSTHs; bin width 20 ms, step size 5 ms) displaying the average net response of responsive neurons in ML (top row), CONTROL (middle row), and AL (bottom row) to the four intact face conditions (examples of these conditions provided at the very top). Blue traces indicate horizontal-passed conditions (solid trace = upright condition; dotted trace = inverted condition), whereas the red traces indicate vertical-passed faces (again, solid trace = upright condition, dotted trace = inverted condition). Stimulus onset corresponds to 0 ms (indicated by a vertical dotted line). Transparent bands reflect the standard error of the mean. (**B**) Smoothed PSTHs (bin width 20 ms, step size 5 ms) displaying the average net response of responsive neurons in ML (top row), CONTROL (middle row), and AL (bottom row) to the four scrambled face conditions (same conventions as in Fig. 2A). (**C**) Distributions of OFI values for the early (black bars) and late (grey bars) response windows in the three regions (from top to bottom ML, CONTROL, and AL).

We analyzed the responses of the 139 ML face-selective neurons (FSI > 0) that responded significantly to the visual conditions (average FSI = 0.56, sd = 0.33; for individual monkey data see Table 1). The 2 × 2 × 2 repeated-measure ANOVA applied to the data in the early time window revealed that, on average, ML neurons responded more in the intact face condition than in the scrambled face condition; F(1,138) = 22.57, p < 0.001). They responded more to upright faces than inverted faces (a classic inversion effect; F(1,138) = 8.48, p = 0.004). There was also a significant interaction between Stimulus Structure and Picture-Plane Rotation (F(1,138) = 6.187, p = 0.014) indicating that there was only evidence of an advantage for upright faces over inverted faces when face structure was intact (Wilcoxon signed-rank test for paired samples, p = 0.001, two-tailed) and not when it was scrambled (Wilcoxon signed-rank test for paired samples, p = 0.253, two-tailed; see Fig. 2A,B) implying that the inversion effect reported previously^18^ was dependent on the presence of facial features in the visual stimuli and not just an oval shape. P-values were corrected for multiple comparisons using the Bonferroni rule.

Averaging across Stimulus Structure and Picture-Plane Rotation there was no evidence that these neurons responded differentially to the orientation filtering conditions (F(1,138) = 3.58, p = 0.06). Instead, Orientation Filtering interacted with Stimulus Structure (F(1,138) = 14.627, p < 0.001). This interaction was driven by a significantly higher response to horizontal-passed faces than vertical-passed faces when the structure of a face was present (Wilcoxon signed-rank test for paired samples (two-tailed), p < 0.001; see Fig. 2A,B). Meanwhile, the same pairwise comparison was not significant when faces were scrambled (Wilcoxon signed-rank test for paired samples (two-tailed), p = 0.58; see Fig. 2A,B). These comparisons were also corrected using the Bonferroni Rule (α = 0.05/2). No other interactions were significant in this analysis (Orientation Filtering*Picture-Plane Rotation, F(1,138) = 3.29, p = 0.07; Stimulus Structure*Orientation Filtering*Picture-Plane Rotation, F(1,138) = 0.84, p = 0.36).Thus, the stronger early response to horizontal-passed than to vertical-passed faces was present for both upright and inverted intact faces in ML.

When the same repeated measures ANOVA was repeated for data collected in the late time window the main effects of Stimulus Structure (F(1,138) = 34.63, p < 0.001) and Picture-Plane Rotation (F(1,138) = 8.09, p = 0.005) were still present. These suggest that neurons were responding more, on average, to intact faces than to scrambled faces and, likewise, more to upright stimuli than inverted stimuli. Although there was no evidence of a main effect of Orientation Filtering (F(1,138) = 0.61, p = .44), we found a significant interaction was between Orientation Filtering and Picture-Plane Rotation (F(1,138) = 4.67, p = 0.03). A subsequent set of pairwise contrasts, adjusted using the Bonferroni rule, indicated that there was a classic inversion effect evident only in the vertical-passed condition (Wilcoxon signed-rank test for paired samples (two-tailed), p = 0.003; see Fig. 2A,B) but not the horizontal-passed condition (p = 0.46). There was no evidence of any other interaction in this analysis.

For intact upright faces, the average OFI value was 0.09 (sd = 0.52) in the early time window, whereas it was −0.07 in the late response window (sd = 0.68). This change during the course of the response in the average preference for orientation-filtered faces from horizontal-passed to vertical-passed faces was significant (Wilcoxon signed-rank test for paired samples (two-tailed), p < 0.001; see Fig. 2C together with Table 1). Meanwhile, there was no evidence that the OFI indices correlated with a neuron’s FSI (*early time window,* N = 139, r = −0.03, p > 0.1; *late time window,* N = 139, r = −0.09, p > 0.1). For the percent of neurons in area ML that were selective for horizontal-passed (OFI > 0.33) or vertical-passed (OFI < −0.33) faces in the early and late time windows see Table 2.

### Area AL

In this region we recorded 137 face-selective neurons (FSI > 0) that were responsive to the Best Identity Experiment (Average FSI = 0.55, sd = 0.31; see Table 1 for individual monkey data). In the early time window, the three-way repeated-measures ANOVA revealed that while AL neurons responded stronger to upright stimuli than to the inverted stimuli (F(1,136) = 15.68, p < 0.001) there was no evidence of a main effect of Stimulus Structure (F(1,136) = 1.12, p = 0.29) or Orientation Filtering (F(1,136) = 3.11, p = .08). Although the interaction between Orientation Filtering and Picture-Plane Rotation was not statistically significant (F(1,136) = 2.83, p = 0.10), there was evidence that Stimulus Structure interacted with both Orientation Filtering (F(1,136) = 5.42, p = 0.02) and Picture-Plane Rotation (F(1,136) = 8.45, p = 0.004). The significant interaction between Stimulus Structure and Orientation Filtering was driven by a stronger response to vertical-passed faces than to horizontal-passed faces only when the structure of the faces was intact (Wilcoxon signed-rank test for paired samples (two-tailed), p = 0.023, Bonferroni corrected; see Fig. 2A,B). There was no evidence of such a bias when faces were scrambled (Wilcoxon signed-rank test for paired samples, p = 0.59, Bonferroni corrected; see Fig. 2A,B). Similarly, two comparisons were used to explain the interaction evident between Stimulus Structure and Picture-Plane Rotation. While AL neurons responded more to upright intact faces than inverted intact faces (Wilcoxon signed-rank test for paired samples, p < 0.001, Bonferroni corrected; see Fig. 2A,B), there was no evidence that picture-plane rotation had the same impact on the strength of their response to scrambled faces (Wilcoxon signed-rank test for paired samples, p = 0.33 (two-tailed), Bonferroni corrected; see Fig. 2A,B).

There was also evidence of a significant three way interaction in the early time window (F(1,136) = 9.84, p = 0.002). To determine the source of this interaction, we ran a discrete series of four Wilcoxon signed-rank tests and corrected their critical values using the Bonferroni rule (α = 0.05/4). These yielded a significant bias towards upright intact faces that were filtered to preserve vertical content when compared to horizontal-passed, upright, intact faces (p = 0.001). No other pairwise comparisons were significant (all p-values > 0.1).

When the data collected in the late time window were analyzed using a 2 × 2 × 2 repeated measures ANOVA, all three main effects were significant. In this later part of the response profile, AL neurons responded significantly more, on average, to intact faces than scrambled faces (F(1,136) = 32.27, p < 0.001), more to vertical-passed faces than horizontal-passed faces (F(1,136) = 7.49, p = 0.007) and more to upright stimuli than inverted stimuli (F(1,136) = 20.06, p < 0.001). Additionally, all resulting interactions were significant; we followed each of these up with a series of two-tailed Wilcoxon signed-rank tests. The significance of all follow up tests was determined after the Bonferroni adjustment. The interaction between Stimulus Structure and Orientation Filtering (F(1,136) = 18.62, p < 0.001) was driven by a stronger average response to vertical-passed, intact faces than to horizontal-passed, intact faces (p < 0.001). The same comparison in the scrambled face data was not significant (p = 0.40). The interaction evident between Stimulus Structure and Picture-Plane Rotation (F(1,136) = 7.92, p = 0.006) was driven by a larger inversion effect (i.e. stronger response to upright stimuli than to inverted stimuli) when faces were intact (p < 0.001) than when faces were scrambled (p = 0.02). Moreover, the interaction between Orientation Filtering and Picture-Plane Rotation (F(1,136) = 7.99, p = 0.005) indicated that the impact of turning stimuli upside down was larger for vertical-passed faces (upright > inverted; p < 0.001) than horizontal-passed faces (upright > inverted; p = 0.08).

As in the early time window, there was also a significant three-way interaction (F(1,136) = 12.84, p < 0.001). To determine its source, we run a series of paired Wilcoxon signed-rank tests and corrected their p-values using the Bonferroni rule (α = 0.05/4). As in the early response window, the results imply that AL neurons responded more to vertical-passed, upright, intact faces than to horizontal-passed, upright, intact faces (p < 0.001). No other comparisons were significant (all p-values > 0.1).

In the intact face data, there was no evidence of a relationship between OFI in the early response window (Average OFI = −0.04, sd = 0.61; see Table 1) and FSI (N = 137, r = −0.02, p > 0.05). Similarly, there was no evidence of a relationship between FSI and OFI in the late response window (Average OFI = 0.20 (sd = 0.64); N = 137, r = −0.04, p > 0.05). A Wilcoxon signed-rank test for paired samples (two-tailed) revealed a significant increase in the average preference for the vertically passed condition, over the vertically passed condition, from the early window to the late response window (p < 0.01).

Interestingly, Fig. 2C reveals that the distribution OFI values for the late response window are very similar for recording regions ML and AL. However, this similarity is not evident in the corresponding PSTHs (see Fig. 2A). One reason for this apparent discrepancy could be that small effects in the later part of the average response in ML were magnified in Fig. 2C when we transformed the data into index values (i.e. normalization). Nonetheless, the differences between recording regions ML and AL are striking; the average late phase of the response in area AL is much stronger and more sustained than in area ML.

### Area CONTROL

112 face-selective (FSI > 0) neurons were recorded in area CONTROL (average FSI = 0.46, sd = 0.45). For data in the early time window, none of the effects were significant (*Stimulus Structure,* F(1,111) = 1.39, p = 0.24; *Orientation Filtering,* F(1,111) = 0.61, p = 0.44; *Picture-Plane Rotation,* F(1,111) = 0.53, p = 0.47; Stimulus Structure*Orientation Filtering, F(1,111) = 0.05, p = 0.82; Stimulus Structure*Picture-Plane Rotation, F(1,111) = 0.98, p = 0.36; Orientation Filtering*Picture-Plane Rotation, F(1,111) = 1.01, p = 0.30; Stimulus Structure*Orientation Filtering*Picture-Plane Rotation, F(1,111) = 1.99, p = 0.16; see Fig. 2A,B).

For data in the late time window, there was a main effect of Stimulus Structure (F(1,111) = 14.33, p < 0.001) indicating that neurons responded more to intact faces than scrambled faces. None of the other effects were significant (*Orientation Filtering,* F(1,111) = 0.03, p = 0.86; *Picture-Plane Rotation,* F(1,111) = 1.33, p = 0.25; Stimulus Structure*Orientation Filtering, F(1,111) = 1.46, p = 0.23; Stimulus Structure*Picture-Plane Rotation, F(1,111) = 0.08, p = 0.78; Orientation Filtering*Picture-Plane Rotation, F(1,111) = 1.31, p = 0.25; Stimulus Structure*Orientation Filtering*Picture-Plane Rotation, F(1,111) = 0.34, p = 0.56; see Fig. 2C)

In area CONTROL there was no evidence that the average OFI value for intact faces in the early response window (M = 0.01, sd = 0.64) differed from the average OFI values in the late response window (M = −0.05, sd = 0.63) using a paired-sampled Wilcoxon signed-rank test (p = 0.53; see Fig. 2C). However, in the early window, there was a weak but significant positive correlation between OFI and FSI (N = 112, r = 0.19, p < 0.05) implying that the neurons with a high FSI tended to be more responsive to horizontal-passed faces relative to vertically passed faces, although this relationship was absent when OFI was calculated on the late time window (N = 112, r = 0.01, p > 0.05).

In light of the relationship between FSI and OFI in the early response window we wanted to rule out the possibility that any differences between the face-selective populations (ML and AL) compared to the CONTROL area were due to a systematic difference in the number of neurons with lower FSI values. We applied a strict FSI criterion to the data in all three regions (FSI > 0.333^27^) thereby selecting only the neurons that responded twice as much to faces as they did to non-face objects in the independent search test. We ran an additional ANOVA on data from the early response window with the following mixed design: *Recording Region* (ML vs. AL vs. CONTROL) x *Upright Face Orientation Filter* (upright horizontal-passed faces vs. upright vertical-passed faces; see Fig. 1B) with the explicit purpose of determining whether the three populations of neurons responded more homogenously to the orientation-filtered conditions when their average face-selectivity was more similar. Under this criterion, the number of neurons in each recording region was reduced (*ML,* N = 97; *AL,* N = 94; *CONTROL,* N = 52), but the average FSI values (*ML,* Average FSI = 0.74; *AL,* Average FSI = 0.71; *CONTROL,* Average FSI = 0.76) did not differ significantly from each other (Kruskal-Wallis ANOVA, p = 0.61). There was no main effect of *Upright Face Orientation Filter* (F(1,236) = 0.41, p = 0.52) but a telling interaction between *Recording Region* and *Upright Face Orientation Filter* (F(2,236) = 8.02, p < 0.001). We confirmed with three Bonferroni corrected contrasts that a significant advantage for horizontal-passed faces over vertical-passed faces in the early response window only occurred in area ML (*ML,* p = 0.003, average OFI = 0.08; *AL,* p = 0.18, average OFI = −0.02; *CONTROL,* p = 0.82, average OFI = 0.005). These results converge to suggest that the positive relationship between FSI and OFI evident among CONTROL neurons, in the early response window, cannot account for the overall result where we found no difference between horizontal-passed faces and vertical-passed faces in that region.

### Multiple Identity Experiment

**Figure 3:**
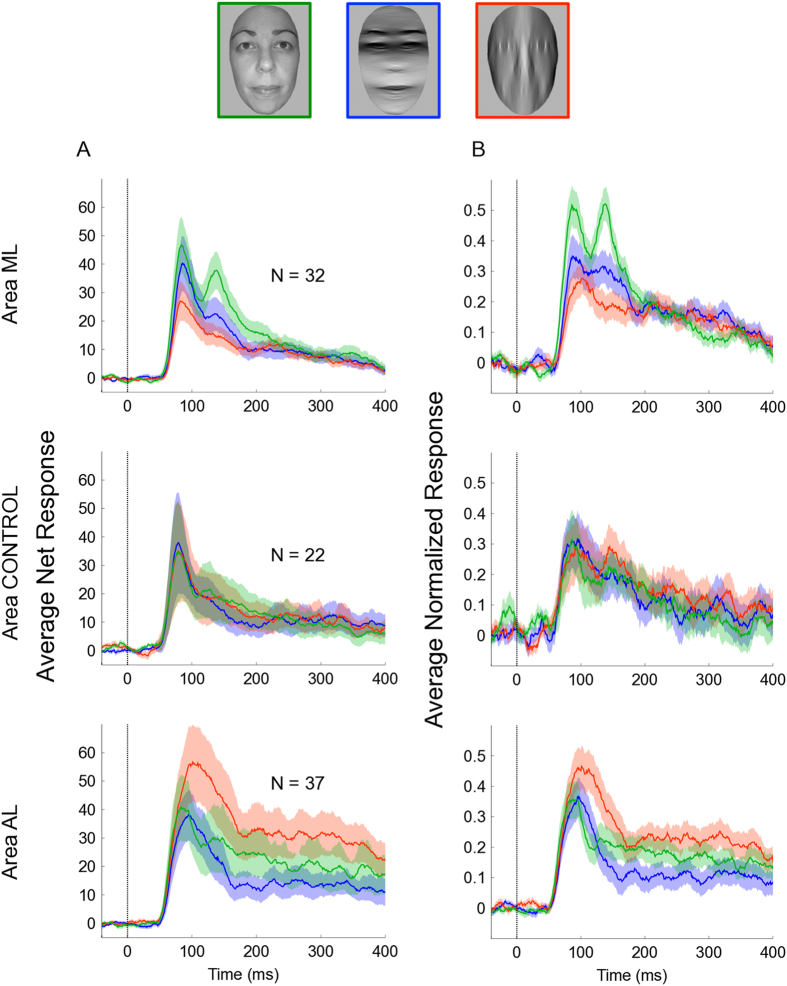
Results from the Multiple Identity Experiment. (**A**) Smoothed PSTHs (same conventions as in Fig. 2A) displaying the average net response of face-selective neurons in ML (top row), CONTROL (middle row), and AL (bottom row) to the three experimental conditions; Green traces indicate the average response of neurons to full spectrum faces, blue traces indicate the average response of neurons to horizontal-passed faces and red traces indicate the average response of neurons to vertical-passed faces. (**B**) Same results as in Fig. 3A but here the data from each neuron was first normalized to its maximum response across the 30 unique stimuli (10 face identities x 3 experimental conditions).

For an independent set of neurons in each region we ran the ‘Multiple Identity Experiment’. The explicit aim here was to investigate single-cell sensitivity for orientation content while avoiding any bias potentially introduced by identity selection. Thus, we presented each neuron with 10 different face identities that were repeated in three different orientation content conditions (full spectrum faces, horizontal-passed faces and vertical-passed faces; see Fig. 3). We presented the 30 unique conditions at least six times per neuron, in pseudo-random order.

Given there is only one factor (Orientation Content) in the Multiple Identity Experiment, and our *a priori* interest in simple pair-wise comparisons between the 3 levels of Orientation Content, we analyzed these data in a series of pairwise contrasts and controlled for multiple comparisons using the False Discovery Rate (FDR) rule^26^, after averaging responses across identity. Under the FDR rule, q(FDR)-values less than 0.05 denote significantly different means, this allowed us to increase the time resolution of the analyses by binning the data into 15 time windows (20 ms in length). To address the concern that, in a small sample size, an effect could be driven by a small number of neurons with a high firing rate, we also analyzed the data after each neuron’s firing rate had been normalized with respect to its maximum response (response computed with a bin width of 300 ms) across all 30 unique conditions. As in the first experiment, we expected to see a stronger response to horizontal-passed faces than to vertical-passed faces. In addition, we expected that the strongest response would be to full spectrum faces.

### Area ML

We recorded the responses of 32 face-selective neurons (FSI > 0) in area ML that responded significantly to the Multiple Identity Experiment (Average FSI = 0.62, sd = 0.34). The average population response (i.e., averaged across the 10 different face identities) was divided into 15 time bins (bin size = 20 ms). In each time bin we tested the difference in average net response to full spectrum faces and horizontal-passed faces, full spectrum faces and vertical-passed faces and, finally, horizontal-passed faces and vertical-passed faces using Wilcoxon signed-rank tests that were corrected using the FDR rule^26^. After correction, there was statistical evidence to suggest that ML neurons, on average, responded more strongly to the full spectrum faces than the horizontal-passed faces in 5 time bins (70–90 ms, 110–130 ms, 130–150 ms, 150–170 ms, and 170–190 ms). They also responded more to full spectrum faces than vertical–passed faces in the same 5 time bins (70–90 ms, 110–130 ms, 130–150 ms, 150–170 ms, and 170–190 ms). Importantly, ML neurons were found to respond more to horizontal-passed faces than vertical-passed faces in the 70–90 ms time bin (see Fig. 3A).

A similar pattern of responses was evident when the data was normalized to each neurons maximum response. After normalization, there was evidence of a stronger response to full spectrum faces than to horizontal-passed faces in four time bins (50–70 ms, 70–90 ms, 130–150 ms and 170–190 ms). There was also evidence that the neurons responded stronger to full spectrum than vertical-passed faces in four time bins (70–90 ms, 110–130 ms, 130–150 ms and 150–170 ms). As with the analysis of the net data, there was one time bin where the ML neurons, collectively, responded more to horizontal-passed faces than to vertical-passed faces but when normalized this significant differential response was delayed (130–150 ms; see Fig. 3B).

### Area AL

We recorded the responses of 37 face-selective neurons in area AL that responded significantly to the Multiple Identity Experiment (Average FSI = 0.54, sd = 0.31). When examining net responses (see Fig. 3A), there was statistical evidence of a stronger response to full spectrum faces than to horizontal-passed faces in 3 time bins (150–170 ms, 190–210 ms and 230–250 ms). There were two successive time bins, beginning at 90 ms after stimulus onset, where there was a stronger response to vertical-passed faces than full spectrum faces. From 110 ms onwards (across 12 time bins) there was also evidence of a much stronger response to vertical-passed faces than to horizontal-passed faces.

After the data was normalized to the maximum response there were only two time bins where the response to full spectrum faces was stronger than to horizontal-passed faces (150–170 ms and 190–210 ms; see Fig. 3B). As in the analysis of the net data, there was a stronger response to vertical-passed faces than full spectrum faces at an early point (90–110 ms). There was also a large continuous period of time, starting 130 ms after stimulus onset, where there was a significantly stronger response to vertical-passed faces than to horizontal-passed faces (130–150 ms, 150–170 ms, 170–190 ms, 190–210 ms, 210–230 ms, 250–270 ms and 310–330 ms). Interestingly, both in net and normalized responses, the differences between conditions emerged later in time course than those evident in area ML (compare Fig. 3A,B).

### Area CONTROL

We recorded the responses of 22 face-selective neurons in the CONTROL area that responded significantly to the Multiple Identity Experiment (Average FSI = 0.36; sd = 0.32). None of the 45 comparisons were significant at the adjusted (FDR) or, even at the unadjusted, level. Furthermore, there was no evidence of any difference between conditions after the data were normalized (also corrected using the FDR correction). Results of this region are presented in Fig. 3A,B.

## Discussion

Here, we investigated how single neurons in three functionally defined IT regions (ML, AL and CONTROL) responded to faces after we had removed all orientation content except horizontal (horizontal-passed) or vertical (vertical-passed) information. We ran two experiments where we presented either the most effective identity (the Best Identity Experiment) or multiple face identities (the Multiple Identity Experiment) to each neuron. We found a different pattern of responses across orientation-filtering conditions, depending on recording region. Neurons in the face patch ML showed the same horizontal content advantage for upright faces as observed in human behavioral and electrophysiological studies, while neurons in the more anterior face patch, area AL, showed the opposite pattern. A population of face-selective neurons outside these face patches did not show any systematic effect of orientation filtering, even when restricting the analysis to neurons that responded twice as much to faces than to nonface objects (FSI > 0.33). The implication is that the face-selective neurons we recorded, despite responding more to face stimuli than non-face objects, may contribute differently to face perception depending on their anatomical location in monkey IT cortex.

When we selected the optimal identity for each neuron (the Best Identity Experiment), face-selective neurons in area ML responded more to horizontal information than vertical when a face’s structure was present, however this effect was only present in the early response window (from 50 until 200 ms). This interaction between Stimulus Structure and Orientation Filtering is consistent with behavioral, electrophysiological, and neuroimaging findings in humans that suggest certain aspects of face perception are supported primarily by horizontal content information^9^,^10^,^11^,^12^,^13^,^15^. Face-selective neurons in this region also responded more to upright faces than inverted faces, an inversion effect consistent with previous reports of single cell activity^18^,^19^,^23^,^27^, but, counter to expectation, there was only an interaction between Orientation Filtering and Picture-Plane Rotation in the late response window and follow up testing revealed this interaction was driven by differential responses in the vertical-passed condition. The expectation that inversion would selectively reduce the response to horizontal-passed faces was based on the observation of such interaction in human data^10^,^11^,^12^. The reason why we do not find such an interaction in area ML remains open to speculation and warrants future investigation. We note that our period of stimulation was quite long and that differential responses in the late time window, especially, might reflect information feeding back into area ML from other regions. Critically, most of the effects reported for intact faces were absent when face structure was removed by phase-scrambling: certainly there was no advantage for horizontal-passed scrambled stimuli over vertical-passed scrambled stimuli. Hence, the observed orientation content sensitivity in area ML was face-specific and independent of external shape, as a grey mask was present in all experimental stimuli.

The early advantage for horizontal-passed faces over vertical-passed faces found in the first experiment, was replicated and extended when we ran the Multiple Identity Experiment on an independent sample of face-selective cells. By averaging across multiple faces that were used without selection, we show the bias towards horizontal-passed stimuli survived changes in stimulus identity. This experiment also showed that, on average, face-selective neurons in area ML respond more to full-spectrum faces than to orientation-filtered faces.

In contrast to the results in area ML, the face-selective neurons in AL responded more to vertical-passed faces than horizontal-passed faces in both experiments. In the Best Identity Experiment, where we selected an optimal identity for each neuron, we found an effect of picture-plane rotation consistent with a previous report of an inversion effect^18^. However, this inversion effect lasted longer in the vertical-passed conditions given that a systematic difference in response strength between upright vertical-passed and inverted vertical-passed conditions was present in both time windows whereas the bias towards upright stimuli in the horizontal-passed condition had disappeared in the late time window. Looking at the time course in Fig. 2A, the data suggest there is an initial transient response to upright horizontal-passed intact faces that is quickly eclipsed by a more sustained response to vertical-passed faces. Again, these effects were not found when we presented the same population of neurons with phase-scrambled faces.

The sustained response to vertical-passed faces in area AL was also replicated in the Multiple Identity Experiment, where we saw an even a stronger response to vertical-passed faces than to their full spectrum equivalents (see Fig. 3A,B). This implies that by selectively preserving the vertical content of a face we enhanced a signal that face-selective neurons in area AL are sensitive to. The exact nature of this signal is an outstanding question that warrants further investigation because very little is known about the role of area AL, as distinct from the roles of other face-selective regions that are close by (i.e. areas AF and AM^1^,^2^,^3^,^4^). It could be that AL neurons are responsible for processing some aspect of the social intention, such as gaze direction, which is arguably more salient in vertically passed faces. It could also be that by applying such a filter, we created the perception of a new stimulus set that appeared more novel in comparison to the full-spectrum or vertically passed faces. This could indicate that area AL is more susceptible to familiarity/novelty effects being perhaps involved in, or sensitive to, stored representations of familiar faces. This could explain the late onset of the vertical orientation content advantage. However, all of these suggestions are still purely speculative and require testing in future experiments.

In sum, we used orientation filtering to investigate the contributions of three distinct populations of neurons in the IT cortex of the rhesus monkey to face perception. By first mapping the IT cortex using an fMRI-localizer, we can conclude that while there are populations of neurons inside the face-selective system are differentially tuned to orientation content, no systematic preference was found among neurons recorded outside the system. Moreover, the only region to show a preference for horizontally passed faces over vertically passed equivalents was the more posterior region, ML, where neurons are selective for viewpoint^6^ and contrast polarity^28^.

## Methods

### Subjects and Localization

We used fMRI to localize the face-selective patches in two male monkeys (*Macaca mulatta*), D and G. All experimental procedures were performed in accordance with the National Institute of Health’s Guide for the Care and Use of Laboratory Animals and EU Directive 2010/63/EU, and approved by the Ethical Committee at the KU Leuven. The animals used in this study (two adult male rhesus monkeys; G&D, 7 kg) were pair-housed with cage enrichment (toys, foraging devices) at the primate facility of the KU Leuven Medical School. They were daily fed with standard primate chow supplemented with bread, nuts, raisins, prunes and fruits. To optimize the signal-to-noise ratio, we used an iron oxide contrast agent (monocrystalline iron oxide nanoparticle or MION (for more details about the procedure^20^). Eighty images of faces, bodies, fruits, manmade objects (gadgets) and hands (16 images per category) were presented to the monkeys in blocks during continuous fixation. These images have been used to isolate face-selective activity both at the system (face-selective patches^1^,^27^) and single unit level (face-selective cells^3^,^27^) in previous studies of rhesus monkeys. Stimuli were presented on a square canvas with a height that subtended a visual angle of 8°. Consistent with previous reports, there were several discrete regions (face-selective patches) in both monkeys that responded more to faces than the four other non-face categories as indicated by the contrast [faces–(bodies + hands + fruits + gadgets)]. More details about the procedure used to localize the face-selective patches in these two subjects have been described elsewhere^18^.

In this study we focus on activity in two face-selective regions in the right hemisphere of both subjects: the middle lateral face patch (ML) and the anterior lateral face patch (AL; see Fig. 1A). ML was located ~4 mm anterior to the interaural line in monkey D and ~6 mm anterior to the interaural line in monkey G. AL was located ~12 and ~13 mm in front of the interaural line in monkeys D and G, respectively. We localized an additional control patch (hereafter referred to as ‘CONTROL’) located between ML and AL patches in both monkeys. The CONTROL patch showed no differential fMRI activation for faces compared to the non-face stimuli in the fMRI data, extending 3 and 2 mm along the anterior-posterior dimension in monkeys D and G, respectively (see Fig. 1A).

### Single Cell Procedure and Analysis

#### Experimental Stimuli

In the single-cell localizer recordings, sixteen images of human faces and sixteen images of non-face objects were used to search for responsive neurons and measure their face selectivity. These images were taken from the image set used during the fMRI localizer. The 16 non-face objects were taken from 4 different categories (headless human bodies, human hands, gadgets, and fruits), selected to be similar to faces in their round shape (e.g. an orange or a closed fist). All images subtended 8° of visual angle in height, the width of the stimuli was allowed to vary. For the electrophysiology, however, the noise background was removed from these images and replaced with a uniform grey background, preserving the natural contour of the objects, and then gamma-corrected.

In the Best Identity Experiment and Multiple Identity Experiment, we employed 24 grayscale pictures of human faces (image subtended 10° of visual angle in height; for illustrative examples of stimuli see previously published work^10^,^12^). These 24 human identities were unfamiliar to the subjects. However, the images were seen numerous times throughout the period of data collection and were all presented in the selection procedure for the Best Identity Experiment, 10 of them were selected for the Multiple Identity Test. The orientation content of these faces was manipulated following the same procedure as in Goffaux and Dakin^10^. Namely, we first subtracted the mean luminance value from each image. Then, we generated filtered stimuli by Fast Fourier transforming the original image using Matlab and multiplying the Fourier energy with a wrapped Gaussian filter centered either on the vertical (0°), or horizontal (90°) orientation with a bandwidth (standard deviation) of 14°. This bandwidth broadly matched the orientation tuning bandwidth of V1 neurons (e.g., Vogels and Orban^29^). Filtering allowed all spatial frequencies to pass within a restricted orientation range. To create the scrambled face stimuli, the phase of the face images was randomly permuted in the Fourier domain, a procedure known to prevent object recognition while preserving its spatial frequency and orientation content^11^,^30^. After the inverse Fourier transform, the global luminance and root-mean square (RMS) contrast of each filtered image was adjusted to match the average global luminance and RMS contrast of the original image set (i.e., luminance: 130 and root mean square contrast: 24 on a scale from 1 to 256 levels of gray). Then the outline mask of the original face images (which was slightly different for each identity) was copied and placed over the corresponding filtered and scrambled images. During single-cell recordings, stimuli were displayed gamma-corrected on a CRT display (Philips Brilliance 202 P4; 1024 × 768 screen resolution; 75 Hz vertical refresh rate) at a distance of 57 cm from the monkey’s eyes.

#### General Procedure

We used the fMRI localizer^17^ to target neurons in three recording regions (ML, CONTROL and AL) using epoxylite-insulated tungsten microelectrodes (FHC) and standard electrophysiological procedures (for more details see Popivanov *et al.*^31^; Sawamura *et al.*^32^). The position of the subject’s right eye was continuously tracked by means of an infrared video-based tracking system (SR Research EyeLink; sampling rate 1 KHz). During the single-cell search test and main experiments, a monkey initiated a trial by fixating on a central fixation spot (size = 0.2° of visual angle) that was present throughout the trial. The monkey was then required to fixate on this spot (within a 2° × 2° fixation window) for 300 ms prior to stimulus onset and during the stimulus presentation (300 ms). An additional 300 ms fixation period after stimulus offset was required before the monkey was rewarded for continuous fixation with a fluid reward. Trials were separated by an inter-stimulus interval of at least 500 ms, the exact duration being dependent on the oculomotor behavior of the monkey in between the trials. All stimuli were presented at the center of the screen, behind the fixation spot. Each trial presented the monkey with a single stimulus, drawn from the set in a pseudo-random order. In each recording session we recorded the first single unit encountered at the predetermined depth with respect to the silence associated with the sulcus, regardless of face-selectivity or visual responsiveness. Each unit thereafter was at least 150 μm deeper than the previous.

After a neuron had been discriminated, we recorded its response to the 32 face and non-face stimuli in the single-cell search test (at least 2 trials per stimulus, thus, a minimum of 64 trials). Firing rate was computed for each unaborted trial in two analysis windows: a baseline window ranging from −250 to 50 ms relative to stimulus onset and a response window ranging from 50 to 350 ms after stimulus onset. Responsiveness of each recorded neuron was tested offline by a split-plot ANOVA with Window (baseline window versus response window) as a repeated-measures factor and Stimulus as a between-trial factor. Only neurons for which either the main effect of the repeated factor or the interaction of the two factors was significant were analyzed further. Following previous studies^4^,^16^, we defined for each neuron a face-selectivity index as FSI = (mean net response faces–mean net response nonface objects)/(|mean net response faces |+| mean net response nonface objects|). The net response corresponds to the firing rate computed in the response window minus the firing rate in the baseline window. Thus, FSI values ranged between 1 and −1, with a value greater than 0 indicating that a unit responded, on average, more to faces than to non-face objects.

#### Best Identity Experiment

The initial identity selection procedure was as follows: we presented each neuron with the 24 intact (i.e. non-scrambled) upright faces that comprised the main experiment stimulus set, sequentially, using the timing parameters described in the general procedure. When all 24 intact faces had been presented at least twice, we selected, online, the face that had elicited the strongest response from the neuron as the effective or “best” face. The best face was then presented in four conditions where face stimuli were ‘intact’ (upright/horizontal-passed, inverted/horizontal-passed, upright/vertical-passed and inverted/vertical-passed) and four conditions where faces were scrambled (upright/horizontal-passed, inverted/horizontal-passed, upright/vertical-passed and inverted/vertical-passed). These eight conditions were repeated at least eight times per neuron (64 trials in total) in pseudo-random order determined programmatically. Visual responsivity was, again, determined offline using a split-plot ANOVA test as described above. There were two additional conditions that appeared intermixed with these eight (upright/no filter and inverted/no filter) these data have been presented elsewhere^18^.

#### Multiple Identity Experiment

For an independent set of neurons in each region we ran the ‘Multiple Identity Experiment’ immediately following the presentation of the face/non-face search stimuli. We presented a neuron with 10 identities that were drawn, at random, from the set of 24 used in the ‘Best Identity Experiment’ and repeated these 10 identities in three different orientation content conditions (full spectrum faces, horizontal-passed faces and vertical-passed faces). We limited the number of conditions included in this experiment in order to make the recording duration of the single units reasonable (because of the large number of identities). For each neuron, the 30 unique conditions were repeated a minimum of six times (180 trials in total) in a different pseudo-random order. Holding neurons for this amount of time would prove more difficult and, thus, the sample sizes for the Multiple Identity Experiment are smaller compared to the first experiment. Offline, we tested for responsivity using a split-plot ANOVA (identical to the procedure described above).

## Additional Information

**How to cite this article**: Taubert, J. *et al.* The impact of orientation filtering on face-selective neurons in monkey inferior temporal cortex. *Sci. Rep.* **6**, 21189; doi: 10.1038/srep21189 (2016).

## Figures and Tables

**Table 1:** Sample sizes and average Index values (with standard deviation) for the Best Identity Experiment.

Region Monkey	ML		CONTROL		AL	
	D	G	D	G	D	G
#of responsive neurons	70	87	42	109	59	91
Average FSI (responsive neurons)	0.37 (0.46)	0.54 (0.41)	0.20 (0.41)	0.23 (0.63)	0.42 (0.38)	0.52 (0.37)
#of face-selective (FSI > 0)neurons	58	81	33	79	52	85
Average FSI (face-selective neurons)	0.52 (0.34)	0.59 (0.33)	0.33 (0.30)	0.51 (0.49)	0.51 (0.30)	0.58 (0.32)
Average OFI (early response window)	0.13 (0.36)	0.05 (0.61)	0.09 (0.58)	−0.02 (0.67)	0.03 (0.53)	−0.09 (0.65)
Average OFI (late response window)	−0.02 (0.64)	−0.11 (0.71)	−0.01 (0.59)	−0.06 (0.65)	−0.09 (0.57)	−0.24 (0.67)

**Table 2:** The % of face-selective neurons with an OFI greater than 0.33 or less than −0.33.

Recording Region	% Face-selective neurons Early window		% Face-selective neurons Late window	
	OFI < −0.33	OFI > 0.33	OFI < −0.33	OFI > 0.33
ML	18	37	49	32
CONTROL	38	39	36	45
AL	39	30	54	21
